# Identification of integrons and gene cassette-associated recombination sites in bacteriophage genomes

**DOI:** 10.3389/fmicb.2023.1091391

**Published:** 2023-01-19

**Authors:** Qin Qi, Vaheesan Rajabal, Timothy M. Ghaly, Sasha G. Tetu, Michael R. Gillings

**Affiliations:** ^1^School of Natural Sciences, Macquarie University, Sydney, NSW, Australia; ^2^ARC Centre of Excellence in Synthetic Biology, Macquarie University, Sydney, NSW, Australia

**Keywords:** integrons, bacteriophages, horizontal gene transfer, mobile genetic elements, gene cassette-associated recombination sites, bacteria–phage coevolution

## Abstract

Bacteriophages are versatile mobile genetic elements that play key roles in driving the evolution of their bacterial hosts through horizontal gene transfer. Phages co-evolve with their bacterial hosts and have plastic genomes with extensive mosaicism. In this study, we present bioinformatic and experimental evidence that temperate and virulent (lytic) phages carry integrons, including integron-integrase genes, *attC*/*attI* recombination sites and gene cassettes. Integrons are normally found in Bacteria, where they capture, express and re-arrange mobile gene cassettes *via* integron-integrase activity. We demonstrate experimentally that a panel of *attC* sites carried in virulent phage can be recognized by the bacterial class 1 integron-integrase (IntI1) and then integrated into the paradigmatic *attI1* recombination site using an *attC* x *attI* recombination assay. With an increasing number of phage genomes projected to become available, more phage-associated integrons and their components will likely be identified in the future. The discovery of integron components in bacteriophages establishes a new route for lateral transfer of these elements and their cargo genes between bacterial host cells.

## 1. Introduction

Bacteriophages are viruses that infect bacteria and are characterized by the sheer diversity, plasticity and mosaic nature of their genomes (Westmoreland et al., 1969; Hershey, 1971; Pedulla et al., 2003; Brüssow et al., 2004; Hatfull and Hendrix, 2011; Mavrich and Hatfull, 2017). As versatile mobile genetic elements, phages play important roles in mediating horizontal gene transfer (HGT), driving the evolution of their bacterial hosts (Zinder and Lederberg, 1952; Lennox, 1955; McDaniel et al., 2010; Villa et al., 2019) and maintaining bacterial population diversity (Buckling and Rainey, 2002; Pal et al., 2007; Rodriguez-Valera et al., 2009; Koskella and Brockhurst, 2014). Genetic materials are exchanged between phages and their bacterial hosts *via* three modes: generalized, specialized and lateral transduction (Zinder and Lederberg, 1952; Lennox, 1955; Parkinson, 2016; Chen et al., 2018; Chiang et al., 2019). Phage-mediated HGT can significantly alter their hosts by increasing their virulence (Wagner and Waldor, 2002), spreading genes that confer new traits, and switching between alternative bacterial lifestyles (Rice et al., 2009; Xia and Wolz, 2014). Phages co-evolve with their bacterial hosts, for example, by acquiring methyltransferases and anti-CRISPR genes that allow them to evade bacterial immune responses (Murphy et al., 2013; Landsberger et al., 2018).

Phages are broadly classified as temperate or virulent (lytic) phages (Lwoff, 1953; Adams, 1959; Howard-Varona et al., 2017; Kirsch et al., 2021). Temperate phages can switch between lytic and lysogenic cycles of replication (Barksdale and Arden, 1974; Cheong et al., 2022). During lysis, phages replicate rapidly in the bacterial cytoplasm before lysing their hosts to release progeny phages that can infect more bacteria (Howard-Varona et al., 2017). During lysogeny, latent phages exist as prophages that are integrated into bacterial genomes. Most prophage genes are thought to be silent, but some genes may be expressed and confer new phenotypes upon their hosts *via* lysogenic conversion (Freeman, 1951; Boyd, 1954; Holloway and Cooper, 1962; Howard-Varona et al., 2017). In contrast, virulent phages, also known more specifically as obligate lytic phages, can only undergo lytic cycles of infection (Brüssow, 2005).

Phages can contribute to the spread of antimicrobial resistance (AMR) genes (Colavecchio et al., 2017; Debroas and Siguret, 2019; Cook et al., 2021), but the extent to which this occurs in natural settings is subject to ongoing debate (Enault et al., 2017; Borodovich et al., 2022). A recent study demonstrated for the first time that AMR genes of class 1 integrons can be found in naturally occurring phage-plasmids that exhibit hybrid properties of both temperate phages and plasmids (Moura de Sousa et al., 2021; Pfeifer et al., 2022). Integrons are genetic elements that can capture, express and re-arrange mobile gene cassettes in diverse bacterial phyla (Stokes and Hall, 1989; Mazel, 2006; Gillings, 2014; Escudero et al., 2015; Ghaly et al., 2020, 2021a). Each complete integron has an integron-integrase gene (*intI*), which encodes a site-specific tyrosine recombinase that catalyses the capture and excision of exogenous gene cassettes into the integron recombination site, *attI* (Hall and Stokes, 1993; Recchia et al., 1994; Escudero et al., 2015). Gene cassettes are circular, non-replicative genetic elements that typically carry an open reading frame (ORF) and a cassette recombination site (*attC*) that can be recombined with *attI* or other *attC* sites in integron gene cassette arrays. Class 1 integrons, which are associated with a highly conserved integron-integrase gene (*intI1*) and a well-defined *attI1* recombination site, are disseminated by mobile genetic elements such as plasmids and transposons (Stokes et al., 1997; Partridge et al., 2000; Gillings et al., 2008). Generally, the spread of class 1 integrons correlates with antimicrobial use and anthropogenic pollution (Gillings et al., 2015; Gillings, 2017). The recent discovery of class 1 integrons in phage-plasmids suggests a previously unrecognized route through which phages can mediate the spread of gene cassette-associated AMR genes (Pfeifer et al., 2022).

In this study, we detect complete integrons or integron components in phage genomes, including examples of CALINs (clusters of *attC*s lacking an associated integron-integrase) in virulent phages that infect environmental bacterial species. Using *attC* x *attI* recombination assays in *Escherichia coli*, we showed that phage-borne *attC* sites can be recognized by IntI1 and integrated into the paradigmatic *attI1* site of class 1 integrons. These results suggest that phage-borne gene cassettes have the potential to be recruited into bacterial integrons and vice versa, which represents an underexplored route of HGT between phages and bacteria.

## 2. Materials and methods

### 2.1. Bioinformatic detection of integrons and *attC* sites in phage genomes

All bacteriophage genomes with the descriptions “complete genomes” or “genomic sequences” were downloaded from the NCBI Genome database (last accessed on May 1, 2022). “Unverified,” “partial” or “incomplete” phage genomes were excluded. IntegronFinder 2.0 was used to detect complete integrons, CALINs (clusters of *attC*s lacking an associated integron-integrase) and In0 elements (integron-integrase that does not carry any gene cassettes) in the 10,705 downloaded phage genomes, using default parameters (Neron et al., 2022). In addition, every phage genome was screened for integron *attC* sites using a previously described HattCI + INFERNAL pipeline (Nawrocki and Eddy, 2013; Pereira et al., 2016; Ghaly et al., 2022a). For the HattCI + INFERNAL analysis, the minimum bit-score threshold was set to 20, and a minimum of two predicted *attC* sites were required in each candidate CALIN according to previously published methods (Ghaly et al., 2022a). All predicted *attC* sites that were located within 3 kB of another *attC* site were used in subsequent analysis. Annotations of *attC* sites were based on the results from both sets of bioinformatic predictions (i.e., IntegronFinder and HattCI + INFERNAL). ORF annotations were based on IntegronFinder results and the original GenBank annotations for the respective phages from NCBI. An in-house script **attC-taxa.sh**^1^ was used to classify the phage-associated *attC* sites according to sequence and structural homologies of chromosomal *attC* sequences in 11 bacterial taxa (Ghaly et al., 2022a).

### 2.2. Construction of *attC* donor strains for *attC* x *attI1* suicide conjugation assays

To confirm that a panel of virulent phage-borne *attC* sites can be recognized by class 1 integron integrase (IntI1) and recombined into the *attI1* site, the bottom strands of six *attC* sites (two each from *T*. *jenkinsii* phage TJE1, *Pseudoalteromonas marina* phage PH101 and *Polaribacter* phage P12002L) were cloned into the mobilisable suicide vector pJP5603 (Riedel et al., 2013). The *attC_aadA7_* is frequently used as a benchmark positive control sequence in *attC* x *attI1* suicide conjugation assays (Bouvier et al., 2005; Biskri et al., 2005; Vit et al., 2021). Overlapping long primers (synthesized by Sigma-Aldrich, United States) were annealed and ligated to the *Xba*I/*Bam*HI restriction sites of pJP5603 (Supplementary Table S3). The ligation products were electroporated into the DAP auxotrophic *E*. *coli* WM3064 λpir strain. The *attC* donor strains were then isolated on LB agar selective media containing kanamycin (Km) and diaminopimelic acid (DAP). To generate *attC* donor plasmids that deliver the top strands of the respective *attC* sites, the same pairs of overlapping long primers were annealed and ligated to the *Xba*I/*Bam*HI restriction sites of the pJP5603rev vector with its *oriT* in an inverted orientation relative to that of the pJP5603 parental vector (Ghaly et al., 2022b).

### 2.3. Quantification of *attC* x *attI1* recombination frequencies

To quantify the recombination frequencies between candidate phage-borne *attC* sites and *attI*, we performed an *attC* x *attI* recombination assay similar to those used in previous works (Bouvier et al., 2005, 2009; Vit et al., 2021). An L-arabinose-inducible, *intI1*-expressing *E*. *coli* recipient strain from a previous study (Ghaly et al., 2022b; UB5201 + pBAD24::*intI1* + pACYC184::*attI1*; carbenicillin (Cb) and chloramphenicol (Cm) resistant) was individually filter-mated in DAP-supplemented LB broth with one of the seven *attC* donor strains (Km resistant; *attC* sequences shown in Supplementary Table S3). IntI1 expression was induced using L-arabinose at 2 mg/ml or suppressed with D-glucose at 10 mg/ml in the negative control set. After 6 h of incubation at 37°C, the resuspended conjugation mix was plated on DAP-free LB agar containing Km. This allowed negative selection of the donor strain (which is not viable in the absence of DAP) and positive selection of the recombinant recipient clones that acquired Km-resistance following co-integration with the *attC* donor plasmids. To quantify the number of potential recipient strain cells, the resuspended conjugation mix was also plated onto DAP-free LB agar containing Cb.

Recombination frequencies were calculated by dividing the colony forming units (CFU) of Km-resistant recombinants by that of the total number of Cb-resistant recipients after 2 days of incubation. The assays were performed in three biological replicates, and recombination frequencies were calculated as the mean of the three independent experiments. One-way ANOVA was used to compare the means of the recombination frequencies for the bottom strands. To verify the sequence of the *attI1*/*attC* recombination junctions, colony PCR was performed on eight randomly chosen colonies per conjugation per biological replicate using GoTaq polymerase (Promega Inc., United States) and the primer pairs pACYC_F (5’-GAACCTTCGAAAAACCGCCC-3′) / M13R (5’-GCGGATAACAATTTCACACAGG-3′) and pACYC_R (5’-ACAGTACTGCGATGAGTGGC-3′) / M13F (5’-TGTAAAACGACGGCCAGT-3′). The thermocycler settings for the colony PCR were as follows: 95°C for 5 min (cell lysis and initial denaturation), 35 cycles of 95°C for 30 s, 55°C for 30 s, and 72°C for 30 s, and 72°C for 5 min. The PCR products were Sanger sequenced for four recombinant colonies per conjugation set (Macrogen Inc., South Korea).

### 2.4. Identification of phages with plasmid replicons

PlasmidFinder (v2.1.6) was used to screen for plasmid replicons in phage genomes that contain integrons or integron components (Carattoli et al., 2014). The BLASTn method with default parameters was used to search the phage genomes against the nucleotide sequences of 116 replicons in the current version of the PlasmidFinder database (last accessed on December 12, 2022).

## 3. Results and discussion

**Figure 1:**
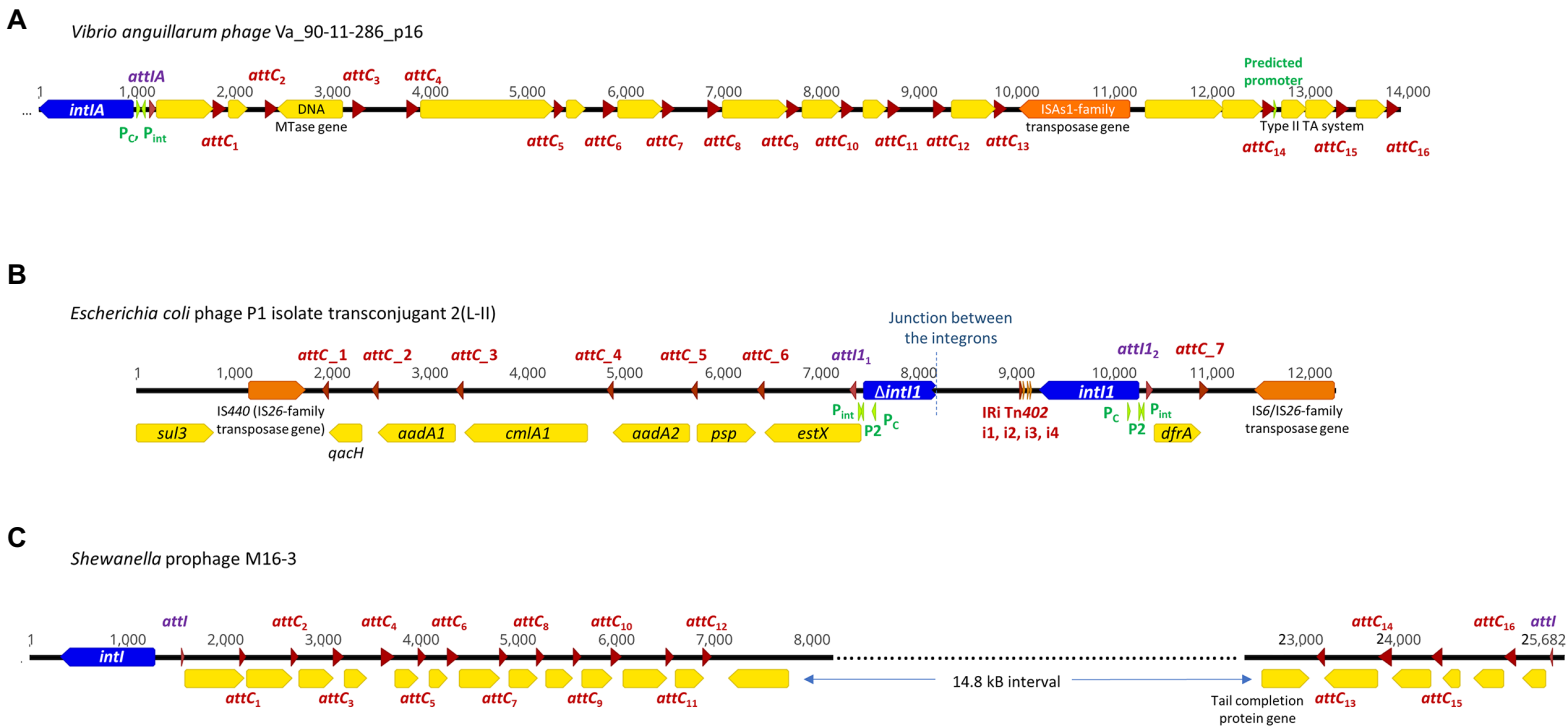
Complete integrons and integron-integrase that does not carry gene cassettes (In0 element) were predicted in four temperate phage genomes. The integron-integrase genes are in blue, the *attC* sites are represented by red arrows, and *attI1* sites by purple arrows. The open reading frames (ORFs) are shown in yellow, and the promoters in green. ORFs encoding predicted DNA methyltransferases (MTase) and toxin-antitoxin modules are specified. Nucleotide numbers are shown above each schematic diagram. **(A)** The functional *V*. *anguillarum* phage Va_90–11–286_p16 carries a complete *Vibrio*-type integron; its gene cassette array is a subset of the chromosomal integron in the host genome. The double-ORF encoding a type II toxin-antitoxin system has its own predicted promoter within that gene cassette. **(B)** *E*. *coli* phage P1 isolate transconjugant 2 (L-II) contains fragments of two class 1 integrons, with their boundary represented by the dotted line. *attC*_1, 2, 4 and 5 can be classified as the *Xanthomonadales* type (Ghaly et al., 2021b). **(C)** *Shewanella* sp. prophage M16-3 contains a complete integron and a CALIN, each with its own *attI* site and integron gene cassette arrays.

**Figure 2:**
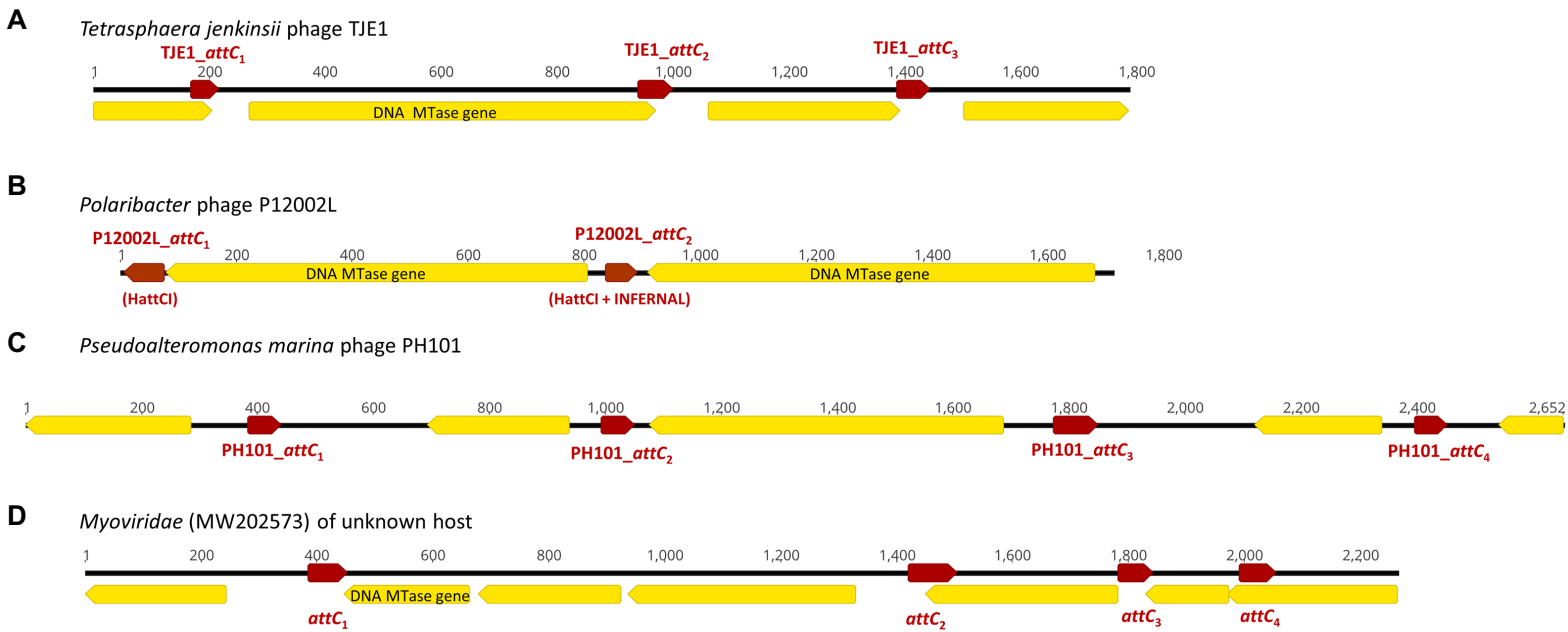
Examples of CALINs found in both virulent and temperate phages with structures that resemble integron gene cassette arrays in bacterial integrons. The open reading frames (ORFs) are shown in yellow, while the *attC* sites are represented by red arrows. ORFs encoding predicted DNA methyltransferases (MTase) are annotated. **(A)** CALIN in the virulent *Tetrasphaera jenkinsii* phage TJE1 contains 4 ORFs and 3 *attC* sites with the typical structure of an integron gene cassette array. **(B)** Two predicted DNA MTase genes were found in the CALIN of the virulent *Polaribacter sp*. phage P12002L. The *attC* sites were predicted with HattCI (P12002L_*attC1*) and HattCI + INFERNAL (P12002L_*attC2*). The latter showed *attC* x *attI1* recombination activity. **(C)** CALIN in the virulent *Pseudoalteromonas marina* phage PH101. **(D)** CALIN in a *Myroviridae* phage (GenBank MW202573) of unknown bacterial host with a putative DNA MTase gene.

We screened 10,705 bacteriophage genomes from the NCBI Genome database for integrons and *attC* recombination sites using previously described IntegronFinder and HattCI + INFERNAL bioinformatic pipelines (Nawrocki and Eddy, 2013; Pereira et al., 2016; Ghaly et al., 2021b; Neron et al., 2022). We detected 3 complete integrons and an integron-integrase without gene cassettes (ln0 element) residing in temperate phage genomes (Table 1), and 17 CALINs obtained from virulent, temperate, and uncharacterized phages (Table 2 and Supplementary Table S1). The genetic contexts of the predicted integrons and CALINs shown in Figures 1, 2 in the phage genomes are summarized in Supplementary Table S2. The annotations for all the predicted integrons and integron components in these phage genomes are available in Supplementary File S1.

**Table 1:** Temperate phage genomes with complete integrons and In0 element (integron-integrase that does not carry gene cassettes) as predicted by IntegronFinder (Neron et al., 2022).

GenBank accession no.	Phage name	Characteristics	References
MK672802	*Vibrio anguillarum* phage Va_90–11–286_p16	41 kB temperate phage inserted as prophage in *V*. *anguillarum* 90–11–286 chromosome II (GenBank CP011461) from rainbow trout (Denmark); SOS-induced lysogenic-to-lytic induction and ability to re-infect other *V*. *anguillarum* strains have been demonstrated	Castillo et al. (2019)
MK672805	*Vibrio anguillarum* phage PF430-3_p42	52 kB temperate phage inserted as prophage in *V*. *anguillarum* PF430-3 (GenBank CP011467) from an unknown fish species (Chile); SOS-induced lysogenic-to-lytic induction and ability to re-infect other *V*. *anguillarum* strains have been demonstrated	
MH445380	*Escherichia coli* phage P1 isolate transconjugant 2 (L-II)	127 kB temperate phage P1 in *E*. *coli* DH5α transconjugant of clinical *E*. *coli* human infant stool strain L-II (Valencia, Spain); a mosaic of two class 1 integrons	Hagbo et al. (2020)
MZ568829	*Shewanella* sp. phage M16-3	40 kB inactive prophage of *Shewanella sp*. M16 (NCBI accession no. NZ_JAGTUL010000000) isolated from biofilm in a gold and arsenic mine (Złoty Stok, Poland)	Bujak et al. (2021)

**Table 2:** Experimentally isolated and characterized virulent phages that contain CALINs with predicted gene cassettes and *attC* sites.

GenBank accession no.	Bacteriophage	Characteristics	*attC* sites for *attC* x *attI1* recombination assay	Reference
NC_019930 (HQ225832)	*Tetrasphaera jenkinsii* phage TJE1	49 kB virulent phage with 66 predicted ORFs; *T*. *jenkinsii* host from activated sludge (Victoria, Australia)	TJE1_*attC*_1_ and *attC*_2_	Petrovski et al. (2012)
NC_028924	*Polaribacter* strain phage P12002L	49 kB virulent phage that infects *Polaribacter* strain IMCC12002; isolated from coastal seawater (Incheon, South Korea); CALIN encodes two putative DNA methyltransferases	P12002L_*attC*_1_ and *attC*_2_	Kang et al. (2015)
NC_029094 (KR534323)	*Pseudoalteromonas marina* phage PH101	132 kB virulent phage with 228 predicted ORFs; *Pseudoalteromonas marina* BH101 host strain isolated from surface seawater (Yellow Sea, China)	PH101_*attC*_1_ and *attC*_2_	Wang et al. (2015)

### 3.1. Discovery of fragments of sedentary chromosomal integrons in fully functional phages of *Vibrio anguillarum*

Integrons can be mobile through their association with mobile genetic elements, such as transposons and plasmids, or sedentary and located in the chromosomes (Loot et al., 2017). Sedentary chromosomal integrons tend to have *attC* sites that are highly conserved in nucleotide sequences and secondary structures (Rowe-Magnus et al., 2003; Ghaly et al., 2021b). The gene cassettes in sedentary chromosomal integrons also tend to carry genes of hitherto unknown functions (Vaisvila et al., 2001; Gillings et al., 2005; Boucher et al., 2007). *Vibrio anguillarum* phages Va_90–11–286_p16 and Va_PF430-3_p42 exemplify the ability of functional temperate phages to capture a segment of integron or integron component from sedentary chromosomal integrons. Their host *V*. *anguillarum* is a Gram-negative pathogen that causes vibriosis in many aquaculture species of fish, crustaceans, and molluscs (Frans et al., 2011). Mitomycin C induced prophage induction has been demonstrated for both phages, which allowed both phages to be purified and subsequently whole-genome sequenced (Castillo et al., 2019). They were further experimentally verified to be functional and can re-infect a variety of other *V*. *anguillarum* strains (Castillo et al., 2019).

Phage Va_90–11–286_p16 (Table 1) is a prophage in chromosome II of the highly virulent *V*. *anguillarum* strain 90–11–286 (Castillo et al., 2017). Intriguingly, its chromosomal excision locations are flanked by two predicted *attC* sites, each of which contains the same 68 bp perfect direct repeat (Supplementary Figure S1A). The location of prophage excision at two *attC* sites could potentially be explained by an increase in integron-integrase activities triggered by the SOS response, which also contributes to prophage induction (Cambray et al., 2011). The direct repeat (DR) at the 5′-end of the prophage insertion site coincides with the 3′-end boundary of a chromosomal CALIN downstream of a truncated integron-integrase gene (Δ*intIA*) in the host chromosome. The DR at the 3′-end of the prophage is found within a sedentary chromosomal integron (SCI) in the host chromosome. The complete integron captured by the prophage is a subset of the much longer sedentary chromosomal integron (SCI) as predicted by IntegronFinder (Neron et al., 2022). Its integron-integrase (IntIA) shares 75% pairwise amino acid identity with that of *V*. *cholera* O1 biovar El Tor strain N16961, while its cassette array comprises mostly single-ORF gene cassettes encoding proteins of unknown functions (Figure 1A). As expected, all the *attC* sites are of the *Vibrionales* type (Ghaly et al., 2021a). A predicted type II RelE/ParE-family endoribonuclease toxin gene and its cognate Phd/YefM-family antitoxin gene are found in a double-ORF operon, which has its own putative promoter. In mobile genetic elements, toxin-antitoxin (TA) systems are primarily considered to be addiction modules that prevent the loss of mobile elements from their host cells (Hayes, 2003). The roles of chromosomal TA systems are more diverse, and less well understood (Hayes, 2003; Saavedra De Bast et al., 2008; Van Melderen, 2010). In the chromosomal integron of *V*. *cholerae*, TA systems contribute to the stabilization of gene cassettes (Iqbal et al., 2015). The *attC* sites found in phage Va_90–11–286_p16 shared a high degree of sequence similarity but were not identical (Supplementary Figure S2).

Phage Va_PF430-3_p42 is a prophage in chromosome II of *V*. *anguillarum* strain PF430-3 and is associated with an integron-integrase without any gene cassettes (In0 element; Supplementary Figure S1B). The IntIA of phage Va_PF430-3_p42 is identical to that of Va_90–11–286_p16 in amino acid sequence. Prophage excision likely occurred at the 5′-end of the integron-integrase (*intIA*) gene in the chromosomal integron of the *Vibrio* host (Supplementary Figure S1B), resulting in the capture of an “orphan” *intIA* gene by the phage.

### 3.2. Mosaic of class 1 integrons in an *Escherichia coli* P1 phage

Unlike most temperate phages, P1 phages exist as circular, autonomous plasmids during lysogeny instead of integrating as prophages into their host chromosomes (Łobocka et al., 2004). A mosaic of two distinct class 1 integrons was found in *E*. *coli* phage P1 isolate transconjugant 2 (L-II; Figure 1B). The clinical *E*. *coli* strain from which this P1 phage originated also harboured IncI_1_, IncF_IB_, and ColE plasmids (Hagbo et al., 2020). Phage P1 isolate transconjugant 2 contains a plasmid replicon that shares 98.6% nucleotide sequence identity with that found in a p0111-type plasmid (NCBI accession no. AP010962; Carattoli et al., 2014). In Figure 1B, the left-hand portion of the map corresponds to the remnant of a *sul3*/IS*440*-associated class 1 integron. Although its IntI1 gene is truncated, all the other features remain intact. The class 1 integron gene cassette array *estX-psp-aadA2-cmlA1-aadA1-qacH* has previously been found in other *Enterobacteriaceae* plasmids of clinical origin (Chah et al., 2010; Chang et al., 2011). The right-hand side of the map in Figure 1B is a complete class 1 integron with an intact *intI1* and a short integron gene cassette array. The non-coding region downstream of *intI1* contains the inverted repeat (IRi) and four associated short repeats (i1-i4) that are characteristic of the Tn*402* transposon, and the 3′-end of gene cassette array has been invaded by an IS*6*/IS*26*-family transposase gene (Kamali-Moghaddam and Sundström, 2001).

Although this P1 phage had transferred from its original *E*. *coli* clinical host strain to a DH5α transconjugant strain, it is not known if it displays all the properties of a functional phage (Hagbo et al., 2020). For a temperate phage to be considered fully functional, it needs to be able to switch from lysogeny to lysis, infect another bacterial cell, repeat the process when infecting a third host cell (Boeckman et al., 2022; Pfeifer et al., 2022). P1 phages are regarded as phage-plasmids that display characteristics of both phages and plasmids. The prevalence of phage-plasmids such as P1, N15 and SSU5 has been estimated to be as high as ~7% amongst all sequenced plasmids (Moura de Sousa et al., 2021). A recent study discovered phage-plasmids carrying class 1 integrons and provided experimental evidence that these phages have the ability to infect/re-infect other bacterial strains and confer AMR phenotypes (Pfeifer et al., 2022). With a growing recognition that many plasmids are in fact phage-plasmids, it is anticipated that more functional and integron-associated temperate phages will be uncovered in the future.

### 3.3. Complete integron and clusters of *attC*s lacking an integron-integrase in an inactive prophage of a *Shewanella* strain

We detected a complete integron and a CALIN in a prophage M16-3 (Figure 1C) in the chromosome of the *Shewanella* species strain M13 (NCBI accession no. NZ_JAGTUL010000000), which was isolated from a gold and arsenic mine. The DNA sequence of M16-3 has been verified by Sanger sequencing (Bujak et al., 2021). Due to a premature stop codon in its phage integrase gene, M16-3 is thought to be a cryptic prophage (Bujak et al., 2021). Integron-associated prophages and prophage-like regions have also been observed in the chromosomes of *K*. *pneumoniae*, *E*. *coli*, and *P*. *aeruginosa* strains (Kondo et al., 2021). In all these cases, it is not clear though if the association between the prophages, complete integrons and/or CALINs occurred before or after the prophages became cryptic.

The intact integron-integrase (IntI) found in phage M16-3 shares 95.3% pairwise amino acid identity with that of *Shewanella oneidensis* MR-1 (GenBank NC_004347; Drouin et al., 2002). A *Shewanella*-type *attI* site (5’-AAACGCGCATGCGCACTAATAAAATGTT-3′), which differs from that of *S*. *oneidensis* MR-1 by a single nucleotide (Drouin et al., 2002), can be found at the start of both the gene cassette array of the complete integron and of the CALIN. Putative phage-specific genes, including those encoding phage tail, capsid scaffolding, and terminase proteins, can be found in the regions flanking the CALIN.

### 3.4. The discovery of clusters of *attC*s lacking an integron-integrase in virulent phages

We discovered 17 CALINs in temperate, virulent, and uncharacterized phages (Table 2 and Supplementary Table S1). Each CALIN in our dataset contains a minimum of two predicted *attC* sites. The gene cassettes in virulent phages *Tetrasphaera jenkinsii* TJE1 and *Pseudoalteromonas marina* PH101 are oriented in the same direction, which is the structure most typical of bacterial gene cassette arrays (Figure 2). We also observed *attC* sites with divergent directions in the *Polaribacter* strain phage P12002L and multiple ORFs between adjacent *attC* sites in the *Myroviridae* phage of unknown host. These observations could potentially be attributed to molecular mechanisms that generate mosaicism in phage genomes.

**Figure 3:**
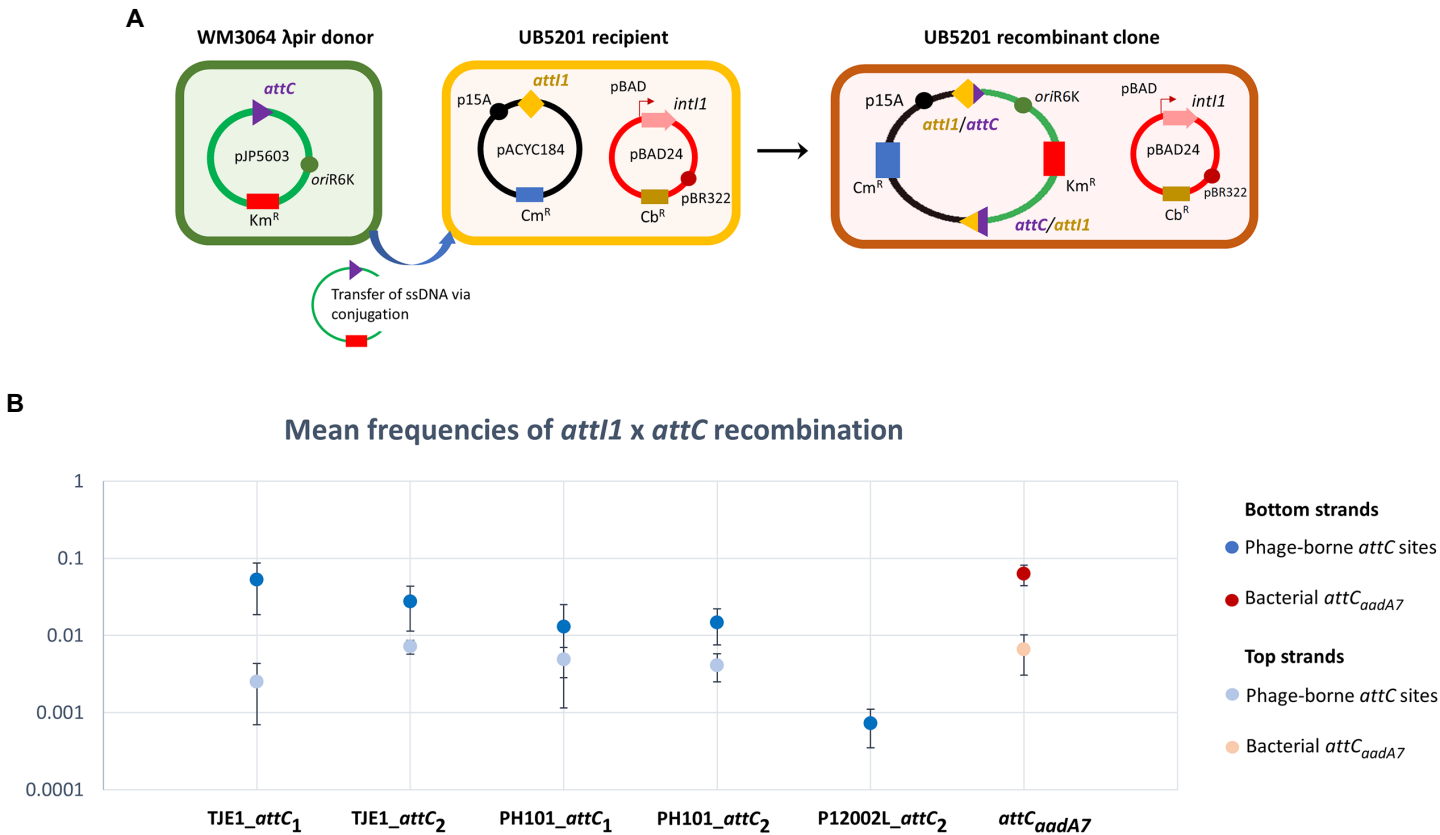
A panel of *attC* sites from virulent phages can be successfully recognized by the class 1 integron-integrase (IntI1) and incorporated into the canonical *attI1* recombination site. **(A)** Schematic of the principles of the *attC* x *attI* recombination assay. During conjugation, the *attC*-containing pJP5603 plasmid is delivered into the UB5201 strain, which carries an L-arabinose inducible *intI1* gene and an *attI1* site on plasmids pBAD24 and pACYC184, respectively. The donor suicide vector cannot replicate within the recipient cells. It can only persist and confer kanamycin resistance following successful *attC* x *attI* recombination to form a co-integrate plasmid in the recombinant clone. **(B)** Mean recombination frequencies (± standard error of the mean) between *attI1* and five virulent phage-borne *attC* sites. The bacterial *attC_aadA7_* site was used as the positive control. Mean recombination frequencies were calculated from three independent sets of biological replicates. No statistically significant difference in mean recombination frequencies was detected across the six successfully integrated bottom strands of *attC* sites (one-way ANOVA: *p* = 0.189, *F*_5, 12_ = 1.79). The mean recombination frequencies of the five successfully integrated top strands of *attC* sites were lower than those of the bottom strands. No recombinant clones were obtained for the top strand of P12002L_*attC_2_*.

**Figure 4:**
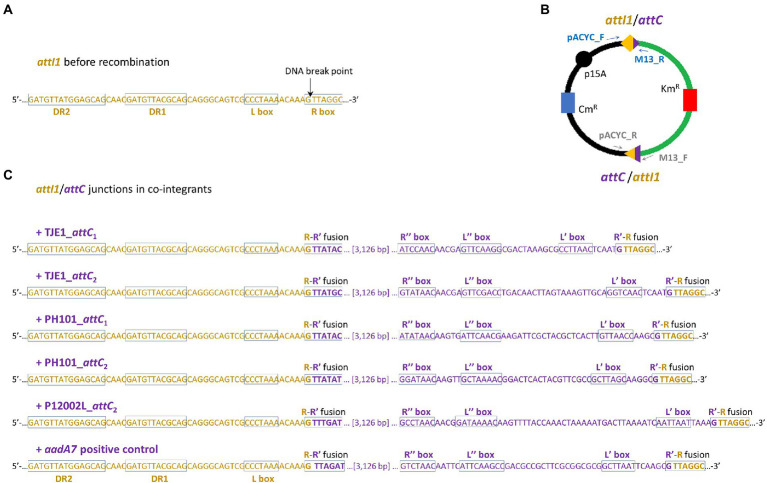
Two *attI1*/*attC* junctions per co-integrant plasmid were formed between the *attI1*-bearing recipient plasmid and the *attC*-donor plasmids. **(A)** DNA sequence of the *attI1* site in the UB5201 recipient strain with L and R boxes (core IntI1 binding sites) and direct repeats DR1 and DR2. DNA cleavage occurs at the insertion points indicated by the black arrows during *attC* x *attI* recombination. **(B)** Schematic of the two recombination junctions in co-integrant plasmids following *attC* x *attI* recombination assay. The two pairs of PCR primers used to verify the two recombination junctions are shown as blue and grey arrows (schematic not drawn to scale). **(C)** DNA sequences at *attC*/*attI* junctions in co-integrant plasmids following *attI1* recombination with five virulent phage-borne *attC* sites and the bacterial *attC_aadA7_* positive control. The nucleotide bases belonging to *attI1* and *attC* are shown in dark orange and purple, respectively. The recombined R-R’ boxes at the two *attC*/*attI* junctions are shown in the rectangles. The inverted repeats of the respective *attC* sites (L’, L,” R’, and R”) are all annotated according to HattCI predictions (Pereira et al., 2016).

We focused on the *attC* sites in three virulent phages that have been isolated from their environmental bacterial hosts for further experimental validation (Table 2; Figures 2A–C). The three bacterial hosts occur in different environments. *T*. *jenkinsii* is a Gram-positive, filamentous bacterial species that causes bulking in activated sludge of wastewater treatment plants (Petrovski et al., 2012). The *Polaribacter* species and *P*. *marina* hosts are found in marine ecosystems such as in surface seawater (Kang et al., 2015; Wang et al., 2015). We demonstrated that five out of the six *attC* sites from these virulent phages can be successfully recognized by the class 1 integron-integrase (IntI1) and incorporated into the canonical *attI1* recombination site. Two adjacent *attC* sites were selected from each phage for their ability to integrate into *attI1* (Table 2). Using a previously described conjugation-based *attC* x *attI1* recombination assay (Bouvier et al., 2005; Nivina et al., 2020; Ghaly et al., 2022b), we showed that gene cassette insertion can take place following the transfer of single-stranded suicide vector DNA with phage-borne *attC* sites (bottom strands) into an *intI1*-expressing *E*. *coli* recipient strain that also harbours an *attI1* site (Figure 3A). With the exception of P12002L_*attC*_1_, the *attI1*/*attC* junctions in the five co-integrant plasmids formed through fusion between the *attI1-*bearing recipient plasmid and the phage-borne *attC* donor plasmids were verified by Sanger sequencing, which confirmed that the correct R-R’ box (5′-G|TTRRRY-3′) sequences were obtained (Figure 4). For the *attC* bottom strands, we found no significant difference in the mean recombination frequencies of the successfully integrated phage-borne *attC* sites and the bacterial *attC_aadA7_* positive control (Figure 3B, one-way ANOVA: *p* = 0.189, *F*_5, 12_ = 1.79). The mean recombination frequencies of the successfully integrated *attC* top strands were lower than those of the respective bottom strands, which is consistent with previous observations that *attC* bottom strands are more recombinogenic than their top strands during *attC* x *attI1* integration (Bouvier et al., 2005; Nivina et al., 2016).

We analyzed the genetic contexts of the predicted integrons/CALINs in the genomes of the phages in Figures 1, 2 and summarized their boundaries in the phage genomes in Supplementary Table S2. Notably, the CALIN in *T*. *jenkinsii* TJE1 was found downstream of a predicted transcriptional terminator (5’-TGTTAGACCGATCGGTCCAAC-3′), which contains two inverted 5’-GTTRRRY-3′ sequences (underlined). *attC* sites are also thought to contain weak transcriptional terminators (Engelstadter et al., 2016). Inverted or multiple 5’-GTTRRRY-3′ motifs can also be seen at the boundaries of the complete integron/CALIN in *Shewanella* sp. phage M16-3. These observations raise the possibility that the similarities between these repeated motifs and the R’/R” boxes that are frequently found in *attC* sites could potentially have contributed to their incorporation into the phage genomes *via* mechanisms such as off-target homologous recombination and illegitimate recombination events (Morris et al., 2008; Bertrand et al., 2019; Pfeifer et al., 2021).

In total, phage-associated gene cassettes carried five predicted DNA methyltransferases (MTases) with significant PFAM matches corresponding to three conserved domains (N6_N4_Mtase, Methyltransf_11 and Methyltransf_16; Supplementary Table S4). In Bacteria, DNA MTases are often accompanied by their cognate restriction endonucleases to form restriction-modification (R-M) systems; restriction endonucleases cleave foreign DNA from invading phages and other mobile genetic elements, while their cognate MTases methylate specific nucleotide bases in bacterial DNA to protect them from DNA cleavage. Some bacterial R-M systems are known to be encoded within integron gene cassettes (Buongermino Pereira et al., 2020; Ghaly et al., 2021a). In temperate and virulent phages, “orphan” DNA MTases that protect phages from bacterial restriction endonucleases have been described (Labrie et al., 2010; Murphy et al., 2013). This observation suggests that phages with integron recombination sites have access to gene cassettes that could confer fitness advantages by overcoming bacterial phage defence mechanisms. Their function is to methylate phage DNA to negate the activity of host encoded restriction endonucleases which recognise the same sequences (Murphy et al., 2014).

In conclusion, we identified integrons and their components in 21 different phages recovered from diverse bacterial host species from different ecosystems. Although the currently observed frequency of association between integrons/integron components and phage genomes is not high, our findings suggest that carriage of integrons in phage genomes could occur across diverse niches and bacterial hosts. While there has been renewed interest in phage biology in recent years, there are still relatively few whole-genome sequenced phages that are available to search for evidence of integrons and CALINs. Additionally, scanning for integrons in prophages from bacterial genome sequences is computationally challenging, since prophage contigs in whole-genome sequencing data are often split by insertion sequences, repetitive sequences and sequences that are homologous to other bacterial genes (Vale et al., 2017; Arndt et al., 2019). With growing interest in phage biology leading to increased rates of genome sequencing, together with improvements in prophage prediction algorithms and long-read DNA sequencing technologies, more integron-associated phage genomes could be identified in the future (Cook et al., 2021).

Here we established that a panel of phage-borne *attC* sites are genuine integron recombination sites that can be recognized and recruited by the bacterial class 1 integron. It has been well demonstrated that integrons and gene cassettes transfer among Bacteria *via* mobile genetic elements such as plasmids and transposons. The finding of operational *attC* sites in virulent phage genomes establishes the potential for phages to also mediate dissemination of integron gene cassettes between bacterial lineages. This mode of HGT has not been fully appreciated and demonstrates that synergies between different types of mobile element could be an important driver of gene transfer.

## Data availability statement

The datasets presented in this study can be found in online repositories. The names of the repository/repositories and accession number(s) can be found at: https://doi.org/10.5061/dryad.z08kprrh5.

## Author contributions

QQ, ST, and MG conceptualization of study. QQ and TG bioinformatic analysis. QQ and VR experimental investigation and validation. MG and ST project management and supervision. QQ writing the original draft of the manuscript. QQ, VR, TG, ST, and MG reviewing and editing the manuscript. All authors contributed to the article and approved the submitted version.

## Funding

This work was supported by the Australian Research Council Discovery Project DP200101874 to MG and ST.

## Conflict of interest

The authors declare that the research was conducted in the absence of any commercial or financial relationships that could be construed as a potential conflict of interest.

## Publisher’s note

All claims expressed in this article are solely those of the authors and do not necessarily represent those of their affiliated organizations, or those of the publisher, the editors and the reviewers. Any product that may be evaluated in this article, or claim that may be made by its manufacturer, is not guaranteed or endorsed by the publisher.

## Footnotes

^1^ https://github.com/timghaly/attC-taxa
