# Supplementary material for: Identification of integrons and gene cassette-associated recombination sites in bacteriophage genomes
Source: Front Microbiol. 2023 Jan 19;14:1091391. doi: 10.3389/fmicb.2023.1091391 (PMC9892861; doi:10.3389/fmicb.2023.1091391)
Supplement: Supplementary file 1 [file Data_Sheet_1.docx]

Supplementary Material

**Table S1**: Fourteen additional temperate, virulent, or uncharacterised phages with predicted integron components that are not shown in Tables 1 and 2.

| **GenBank accession no.** | **Bacteriophage** | **No. of predicted *attC* sites** | **Characteristics** | **Reference** |
| --- | --- | --- | --- | --- |
| MK448889 | *Streptococcus ictaluri* phage Javan262 | 2 | 23 kB temperate phage; prophage identified by PhageMiner in *Streptococcus ictaluri* 707-05 strain from channel catfish with osteolytic bone lesions (Mississippi, United States) | [1, 2] |
| MK448672 | *Streptococcus dysgalactiae* phage Javan117 | 3 | 40 kB temperate phage; prophage identified by PhageMiner in *Streptococcus dysgalactiae* *ssp.* *equisimilis* ASDSE_99 strain from human patient (United States) | [1, 3, 4] |
| MN013086 | *Klebsiella pneumoniae* phage vB_Kpn_Chronis | 2 | 46 kB temperate phage with 73 ORFs with close relatives in *Klebsiella pneumoniae* genomes; experimentally isolated from wastewater strain (United States) | [5] |
| MW202573 | *Myoviridae* phage of unknown bacterial host | 4 | 35 kB *Myoviridae* phage of unknown host isolate ctTUS972 from Suwannee River water sample (Florida, United States); likely to be virulent phage | [6] |
| MH572402 | *Microviridae* phage of unknown bacterial host | 2 | 4 kB *Microviridae* phage of unknown host isolate SD_SF_47 from marine invertebrate *Ciona robusta* (California, United States) | [7] |
| HQ632860 | *Vibrio* *splendidus* phage jenny 12G5 | 2 | 41 kB virulent phage isolated from *Vibrio splendidus* 12G05 strain found in coastal seawater (Massachusetts, United States) | [8] |
| KT160311 | *Vibrio* *kanaloae* phage H188 | 2 | 50 kB virulent phage isolated from *Vibrio kanaloae* in surface seawater (Yellow Sea, China) | [9] |
| KX119177 | *Helicobacter pylori* phage Sw-A626-G | 3 | 31 kB prophage predicted by PHAST; *Helicobacter pylori* strain from gastritis patient (Sweden) | [10, 11] |

**Table S1** (continued)

| **GenBank accession no.** | **Bacteriophage** | **No. of predicted *attC* sites** | **Characteristics** | **Reference** |
| --- | --- | --- | --- | --- |
| KX119202 | *Helicobacter* *pylori* phage Pt-1293-U | 2 | 30 kB prophage predicted by PHAST; *Helicobacter pylori* strain from peptic ulcer patient (Portugal) |  |
| MN855629 | *Myoviridae*-like phage isolate 471 (unknown species) | 2 | 7 kB *Myoviridae*-like phage of unknown host in honeybee metagenome (Belgium); likely to be virulent phage | [12] |
| MT066160 | *Vibrio* *cholerae* phage Saratov-12 | 2 | 48 kB phage from environmental *Vibrio cholerae* strain (Saratov, Russia) | Unpublished |
| NC_025466  (KJ018214) | *Shewanella* *sp.* phage 3/49 | 2 | 40 kB virulent phage isolated from *Shewanella* species strain found on sea ice (Baltic Sea, Finland) | [13] |
| NC_028933  (KP233880) | *Pseudomonas aeruginosa* phage PhiCHU | 2 | 46 kB virulent phage isolated from *Pseudomonas aeruginosa* (Russia) | [14] |
| NC_047845  (MF403005) | *Agrobacterium* *tumefaciens* phage Atu_ph02 | 2 | 54 kB phage found in *Agrobacterium tumefaciens* C58 strain (United States) | Unpublished |

**Table S2**: The genetic contexts of the predicted integrons and CALINs in the phage genomes. The 5’ and 3’-ends are defined according to the orientation the integrons/CALINs are displayed in Figures 1, 2 as well as in Supplementary File 1. Upstream or downstream regions of *intI*, *attI* or outermost *attC* sites are shown. Unless otherwise mentioned, each figure shows a 500 bp region at the specified boundary.

| 0BPhage name | 1B5’/3’- end | 2BBoundaries of integrons and CALINs in phage genomes |
| --- | --- | --- |
| 3B*V. anguillarum* phage Va_90-11-286_p16 | 4B5’ | 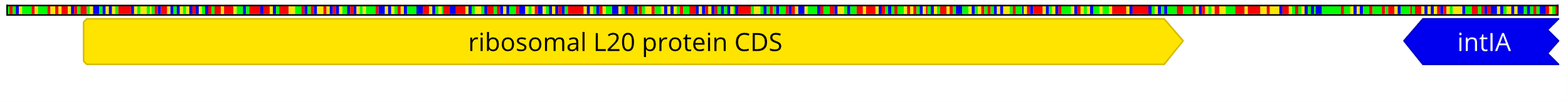 |
|  | 5B3’ | 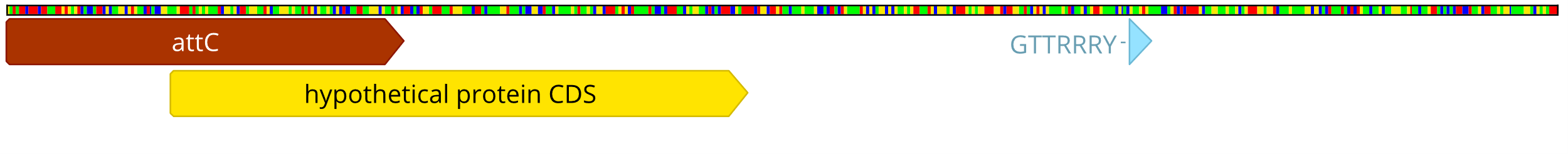 |
| 6B*E. coli phage P1 isolate transconjugant 2 (L-II)* | 7B5’ | 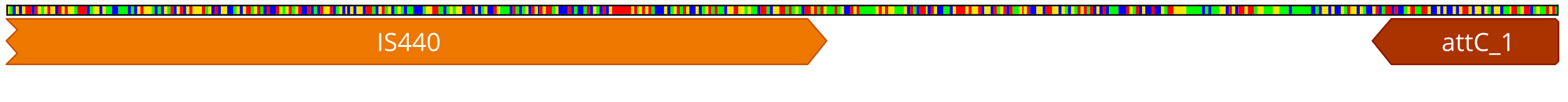 |
|  | 8B3’ | 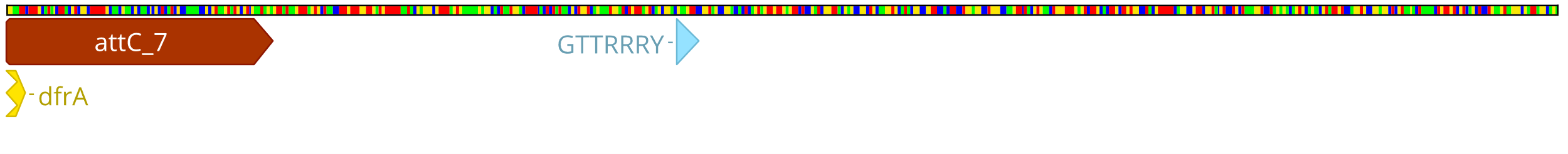 |
| 9B*Shewanella* sp. phage M16-3 | 10B5’ | 11BBoundary of complete integron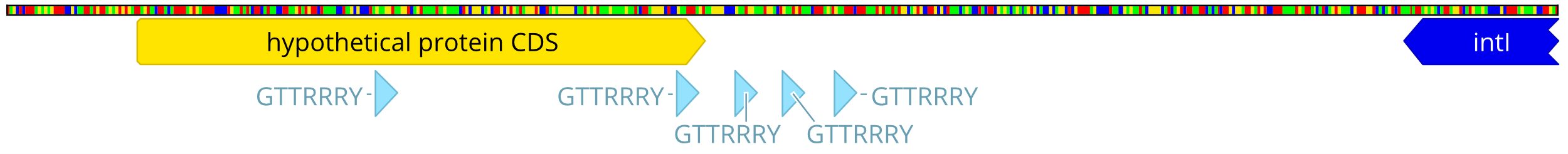 |
|  | 12B3’ | 13BBoundary of complete integron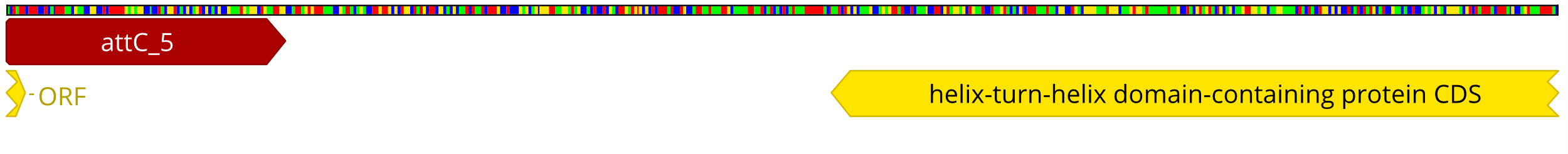 |
|  | 14B5’ | 15BBoundary of CALIN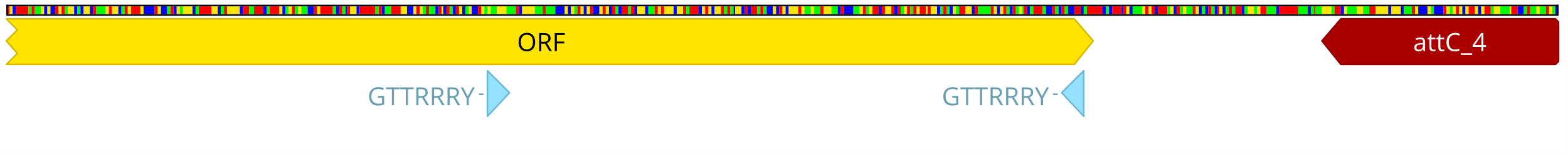 |
|  | 16B3’ | 17BBoundary of CALIN 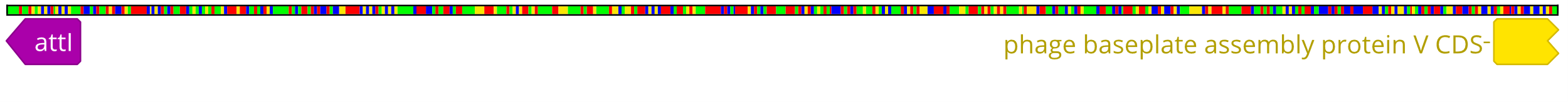 |
| 18B*T.*  *jenkinsii* phage TJE1 | 19B5’ | 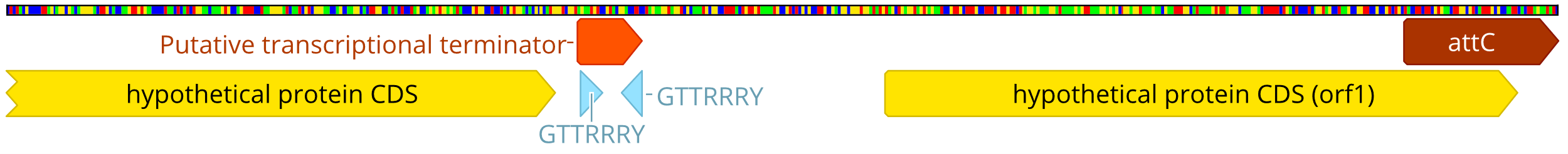 |
|  | 20B3’ | 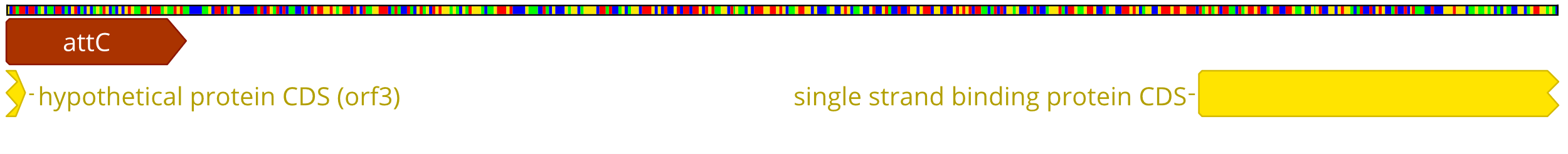 |
| 21B*Polaribacter* strain phage P12002L | 22B5’ | 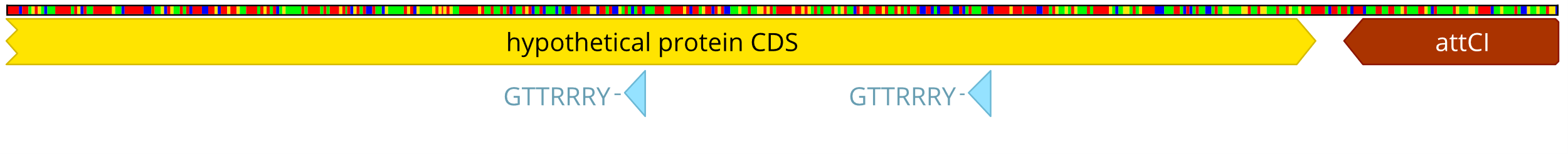 |
|  | 23B3’ | 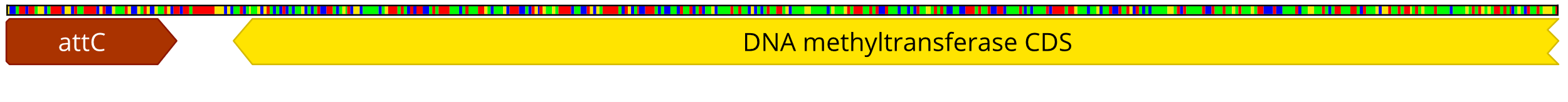 |

**Table S2** (continued)

| 0BPhage name | 1B5’/3’- end | 2BBoundaries of integrons and CALINs in phage genomes |
| --- | --- | --- |
| 24B*P. marina* phage PH101 | 25B5’ | 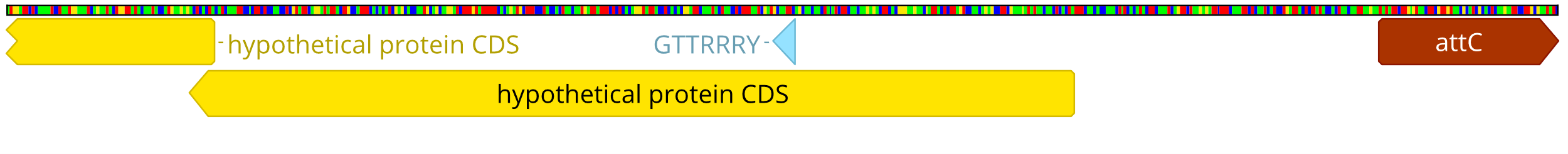 |
|  | 26B3’ | 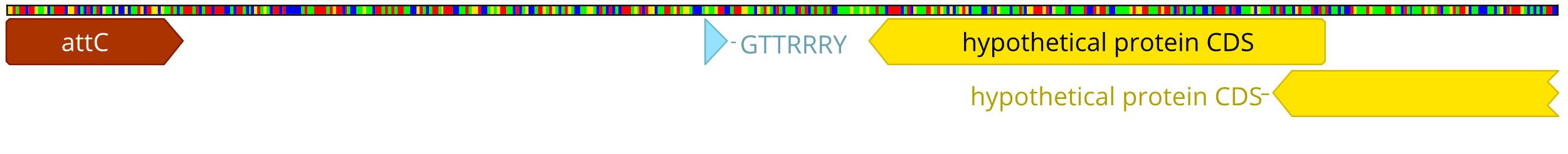 |
| 27B*Myoviridae* phage of unknown host ctTUS972 | 28B5’ | 29BOnly 453 bp shown due to short phage length: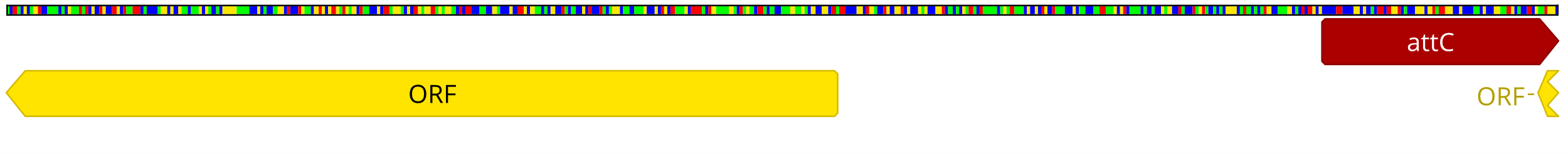 |
|  | 30B3’ | 31BOnly 273 bp shown due to short phage length: 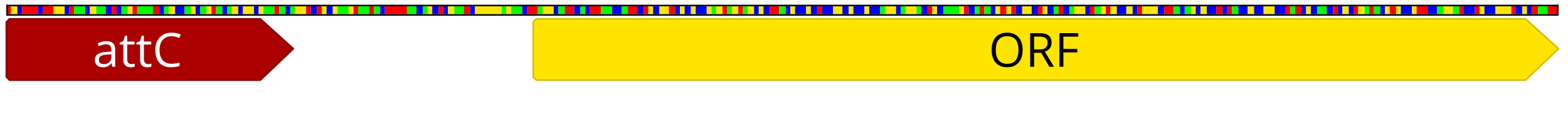 |

**Table S3**: Pairs of overlapping primers used to generate the pJP5603::*attC* and pJP5603rev::*attC* suicide vectors carried by the *E. coli* WM3064 λpir donor strains. The nucleotide sequences of the bottom strands (5’->3’) of the *attC* sites are shown in green.

| ***attC* site** | **Source** | **Primer** | **Oligonucleotide sequence (5’->3’)** |
| --- | --- | --- | --- |
| *aadA7* positive control | R388 IncW plasmid with class 1 integron | Forward | CTAGAATGTGCTTAGTGCATCTAACGCTTGAATTAAGCCGCGCCGCGAAGCGGCGTCGGCTTGAATGAATTGTTAGACATCG |
|  |  | Reverse | GATCCGATGTCTAACAATTCATTCAAGCCGACGCCGCTTCGCGGCGCGGCTTAATTCAAGCGTTAGATGCACTAAGCACATT |
| TJE1_*attC*_1_ | *T. jenkinsii* phage TJE1 | Forward | CTAGAGTATAACATTGAGTTAAGGCGCTTTAGTCGCCTTGAACTCGTTGTTGGATG |
|  |  | Reverse | GATCCATCCAACAACGAGTTCAAGGCGACTAAAGCGCCTTAACTCAATGTTATACT |
| TJE1_*attC*_2_ |  | Forward | CTAGAGCATAACATTGAGTTGACCTGCAACTTTACTAAGTTGTCAGGTCGAACTCGTTGTTATACG |
|  |  | Reverse | GATCCGTATAACAACGAGTTCGACCTGACAACTTAGTAAAGTTGCAGGTCAACTCAATGTTATGCT |
| PH101_*attC*_1_ | *P. marina* phage PH101 | Forward | CTAGAGTATAACGCTTGGTTAACAAGTGAGCGTAGCGAATCTTCGTTGAATCACTTGTTATATG |
|  |  | Reverse | GATCCATATAACAAGTGATTCAACGAAGATTCGCTACGCTCACTTGTTAACCAAGCGTTATACT |
| PH101_*attC*_2_ |  | Forward | CTAGAATATAACGCCTTGCTAAGCGGCGAACGTAGTGAGTCCGTTTTAGCAACTTGTTATCCG |
|  |  | Reverse | GATCCGGATAACAAGTTGCTAAAACGGACTCACTACGTTCGCCGCTTAGCAAGGCGTTATATT |
| P12002L_*attC*_1_ | *Polaribacter* *sp.* phage P12002L | Forward | CTAGATGCTAACGGCTACGGCTAAACGGTCGTTTTAATGCCGTTTTAGCCATTGTTAGGCG |
|  |  | Reverse | GATCCGCCTAACAATGGCTAAAACGGCATTAAAACGACCGTTTAGCCGTAGCCGTTAGCAT |
| P12002L_*attC*_2_ |  | Forward | CTAGAATCAAACTTTAATTAATTGATTTTAAGTCATTTTTAGTTTGGTAAAACTTGTTTTATCCGTTGTTAGGCG |
|  |  | Reverse | GATCCGCCTAACAACGGATAAAACAAGTTTTACCAAACTAAAAATGACTTAAAATCAATTAATTAAAGTTTGATT |

**Table S4**: Predicted DNA methyltransferases that are encoded by five phage-borne gene cassettes. Domains with significant PFAM matches (*E-*value < 0.005) in their amino acid sequences are underlined.

| **Phage** | **Amino acid sequence (N- to C-terminus)** | **PFAM domain** | ***E*-value** |
| --- | --- | --- | --- |
| *V. anguillarum* phage Va_90-11-286_p16 | MTTTLRIRYQTIVIGNNDIHLCTLRDKQQFNDPKNTAQNLGISSASWPIFGVVWPSSLVLAHHVLNLDTNNKRILEVGCGIGLSSLLLNEQMANITATDYHPEVETFLNRNTPLNNRKKIAFERVDWADANSQLGLFDLIIGSDLLYEDQHTSLLAQFIQTHANPTCDIIIVDPDRGRKNKLSAKMSEYGFTSDHIRPDNTDYLEQKFKGHILRFSRTSESI | Methyltransf_16  (PF10294); lysine methyltransferase | 8.8e-15 |
| *T. jenkinsii* phage TJE1 | MPTSRKGEKMVEMLNCDCMGYMATLSDKAFELAIVDPPYGIGAGDVKRGGQQHGNALAPSKAYEKKEWDSMPPPQEYFDELFRVSKNQIIWGGNYYSLPVSSAWVFWDKVTGNNGYADGELAWTSFESALRMFRFEWHGMLQGNMKNKESRIHPTQKPVALYKWILSRYAKPGDKILDTHGGSGSICIACHDLGYDLTWMELDADYYEAACKRYKDHAAQAVLFEPQEIRQKTYNNEFDLTT | N6_N4_Mtase (PF01555);  N-4 cytosine- or N-6 adenine-specific methyltransferase | 1.3e-10 |
| *Polaribacter* *sp.* phage P12002L | MIDIRLSDNLDLMAEMQDNTVDLIYCDILYGTGRKFKDYQDLKPKREIIEEHYIPRIKEMHRVLKDTGSIYLQMDSKINHWMRCILDDVFGYDMFLCEIIWVYGSGLKAKSKKFHTHNDSILHYTKENKHTYNPQMIKLDKPSKRWVTNTINGKNVPKRDANGKQETYFVKEIKVGNIWKDIPMRRNGIIYQTEKPKALIERIIKASSNEGDLVADFYAGSFTTAEVCKDLNRNFIGCDISEKAVQIGRARVRSQQ |  | 1.4e-40 |
| *Polaribacter* *sp.* phage P12002L | MIDLRLGDCLEVMKTIEDKSIDAIICDLPYGTTDCKWDSIIQFDKLWPQYERIIKDNGAIVLFGAEPFSSLLRCSNLNIYKYDWIWDKVNKKVGYGNAKRQPLRGVENISVFYKKQCTYNPIMGVGKPYKSKSGGNTKIYEKGGLKSIVTDNKGTRYPHSIISIKGDLKKEMGLHPTQKPVELLEYLIKTYTNDGETVLDNTMGSGSTGVACKQTNRKFIGIEREEKYFKIAQERINSTLF |  | 5.9e-26 |
| *Myoviridae* of unknown host ctTUS972 (MW202573) | MPPKVVGSGKDARVRKCLPDRVHDFRDMAYPDETFQMVVFDPPHLFLGENSFMAQSYGRLDKETWKEDLSRGFGECFRVLKTGGVLIFKWNECDIPLAEILKCAQHQPLFGHPSGKAQKTHWCAFMKPNV | Methyltransf_11 (PF08241); SAM-dependent methyltransferases | 0.0033 |


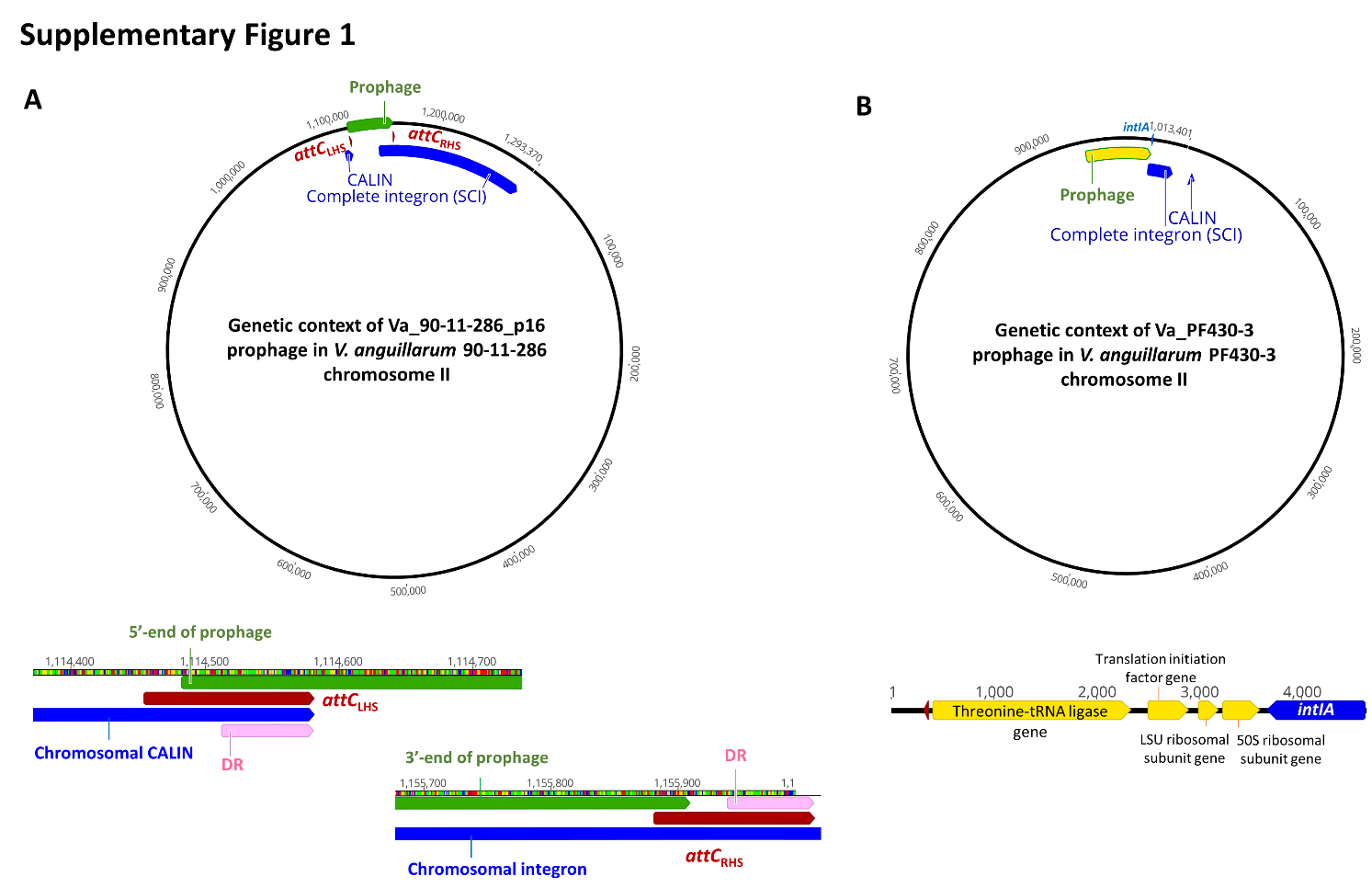


**Supplementary Figure 1: Genetic context of *V. anguillarum* prophages Va_90-11-286_p16 and Va_PF430-3_p42 in their respective host chromosomes.**

**(A)** During SOS-induced lysogeny to lysis transition, the Va_90-11-286_p16 prophage (in green) was excised from two chromosomal *attC* sites (in red); one on the left (*attC*_LHS_), and one on the right-hand side (*attC*_RHS_). Both sites contain a perfect 68 bp direct repeat (DR). The prophage insertion sites at the 5’- and 3’-ends of Va_90-11-286_p16 are shown in the inset. The chromosomal integrons and CALINs in the host chromosomes are displayed in dark blue. **(B)** *V. anguillarum* phage PF430-3_p42 contains a putative In0 element with neither an *attIA* site nor a gene cassette array. The neighboring prophage genes (in yellow) upstream of the In0 element are shown in the inset.

**
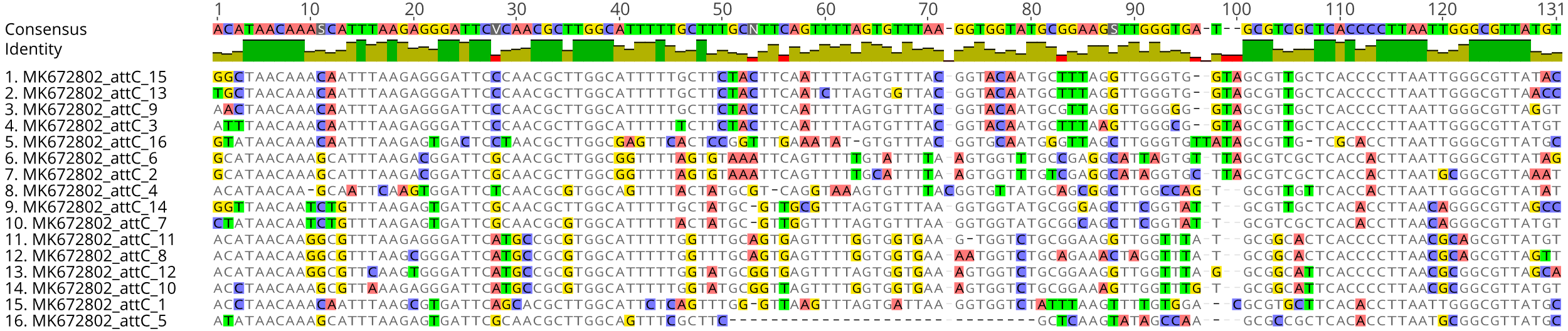
**

**Supplementary Figure 2: *attC* sites in the *V. anguillarum* prophage Va_90-11-286_p16 show a high degree of nucleotide sequence similarity.** Nucleotide sequence alignment of all the 16 *attC* sites found in the *V. anguillarum* prophage Va_90-11-286_p16 (cost matrix: ClustalW; gap open cost = 15; gap extend cost = 6.66) showed that the average identity over the alignment was 67.5%.

**Supplementary File 1**: Annotations of complete integrons, CALINs, and In0 elements in 21 phage genomes predicted by IntegronFinder 2.0 in GenBank format. The file is available on the Dryad Data Repository: <https://doi.org/10.5061/dryad.z08kprrh5>

**References for Supplementary Material**

1. Rezaei Javan, R., et al., *Prophages and satellite prophages are widespread in Streptococcus and may play a role in pneumococcal pathogenesis.* Nat Commun, 2019. **10**(1): p. 4852.

2. Richards, V.P., et al., *Phylogenomics and the dynamic genome evolution of the genus Streptococcus.* Genome Biol Evol, 2014. **6**(4): p. 741-53.

3. Sichtig, H., et al., *FDA-ARGOS is a database with public quality-controlled reference genomes for diagnostic use and regulatory science.* Nat Commun, 2019. **10**(1): p. 3313.

4. Hawke, J.P., et al., *Streptococcus dysgalactiae: A pathogen of feral populations of silver carp from a fish kill event.* J Aquat Anim Health, 2021. **33**(4): p. 231-242.

5. Thurgood, T.L., et al., *Genome sequences of 12 phages that infect Klebsiella pneumoniae.* Microbiol Resour Announc, 2020. **9**(16).

6. Malki, K., et al., *Spatial and temporal dynamics of prokaryotic and viral community assemblages in a lotic system (Manatee Springs, Florida).* Appl Environ Microbiol, 2021. **87**(18): p. e0064621.

7. Creasy, A., et al., *Unprecedented diversity of ssDNA phages from the Family* *Microviridae detected within the gut of a Protochordate model organism (Ciona robusta).* Viruses, 2018. **10**(8).

8. Thompson, J.R., et al., *Genotypic diversity within a natural coastal bacterioplankton population.* Science, 2005. **307**(5713): p. 1311-3.

9. Li, Y., et al., *Complete genomic sequence of bacteriophage H188: A novel Vibrio kanaloae phage isolated from Yellow Sea.* Curr Microbiol, 2016. **72**(5): p. 628-33.

10. Vale, F.F., et al., *Genomic structure and insertion sites of Helicobacter pylori prophages from various geographical origins.* Sci Rep, 2017. **7**: p. 42471.

11. Arndt, D., et al., *PHAST, PHASTER and PHASTEST: Tools for finding prophage in bacterial genomes.* Brief Bioinform, 2019. **20**(4): p. 1560-1567.

12. Deboutte, W., et al., *Honey-bee-associated prokaryotic viral communities reveal wide viral diversity and a profound metabolic coding potential.* Proc Natl Acad Sci U S A, 2020. **117**(19): p. 10511-10519.

13. Senčilo, A., et al., *Cold-active bacteriophages from the Baltic Sea ice have diverse genomes and virus-host interactions.* Environ Microbiol, 2015. **17**(10): p. 3628-41.

14. Krylov, V., et al., *Selection of phages and conditions for the safe phage therapy against Pseudomonas aeruginosa infections.* Virol Sin, 2015. **30**(1): p. 33-44.
